# SAF-A mutants disrupt chromatin structure through dominant negative effects on RNAs associated with chromatin

**DOI:** 10.1007/s00335-021-09935-8

**Published:** 2021-12-02

**Authors:** Heather J. Kolpa, Kevin M. Creamer, Lisa L. Hall, Jeanne B. Lawrence

**Affiliations:** 1 Department of Cell and Developmental Biology, University of Massachusetts Medical School, Worcester, MA 01655 USA; 2 Present Address: Ashfield MedComms, Lyndhurst, NJ 07071 USA; 3 Present Address: Department of Neurology, University of Massachusetts Medical School, Worcester, MA 01655 USA

## Abstract

Here we provide a brief review of relevant background before presenting results of our investigation into the interplay between scaffold attachment factor A (SAF-A), chromatin-associated RNAs, and DNA condensation. SAF-A, also termed heterogenous nuclear protein U (hnRNP U), is a ubiquitous nuclear scaffold protein that was implicated in XIST RNA localization to the inactive X-chromosome (Xi) but also reported to maintain open DNA packaging in euchromatin. Here we use several means to perturb SAF-A and examine potential impacts on the broad association of RNAs on euchromatin, and on chromatin compaction. SAF-A has an N-terminal DNA binding domain and C-terminal RNA binding domain, and a prominent model has been that the protein provides a single-molecule bridge between XIST RNA and chromatin. Here analysis of the impact of SAF-A on broad RNA-chromatin interactions indicate greater biological complexity. We focus on SAF-A’s role with repeat-rich C_0_T-1 hnRNA (repeat-rich heterogeneous nuclear RNA), shown recently to comprise mostly intronic sequences of pre-mRNAs and diverse long non-coding RNAs (lncRNAs). Our results show that SAF-A mutants cause dramatic changes to cytological chromatin condensation through dominant negative effects on C_0_T-1 RNA’s association with euchromatin, and likely other nuclear scaffold factors. In contrast, depletion of SAF-A by RNA interference (RNAi) had no discernible impact on C_0_T-1 RNA, nor did it cause similarly marked chromatin changes as did three different SAF-A mutations. Overall results support the concept that repeat-rich, chromatin-associated RNAs interact with multiple RNA binding proteins (RBPs) in a complex dynamic meshwork that is integral to larger-scale chromatin architecture and collectively influences cytological-scale DNA condensation.

**Supplementary Information:**

The online version contains supplementary material available at 10.1007/s00335-021-09935-8.

## Introduction

Interphase chromosomes are associated with a diverse abundance of RNAs, ranging from highly produced pre-mRNAs to thousands of low-level lncRNAs, and evidence increasingly suggests this collection of chromatin-associated hnRNA broadly influences large-scale nuclear chromosome architecture. XIST RNA is the preeminent example of an RNA whose influence on local chromatin packaging involves recruiting histone modifying enzymes (Brockdorff et al. 2020; Creamer and Lawrence 2017; Loda and Heard 2019). However, a distinct concept considered here and bolstered by recent evidence (Creamer et al. 2021) is that the physical presence of RNA can also more directly impact chromosome territory structure, likely by interaction of long nascent RNAs with a network of insoluble structural proteins. Here we focus on SAF-A, which has been suggested to have a broad effect on nuclear euchromatin structure but was also implicated to support localization of XIST RNA to Xi heterochromatin. To provide fuller context, as suggested by the editors, we first provide a brief review of multiple areas that intersect in the complex biology illuminated here. We then present unpublished work in which we investigated how a series of perturbations of SAF-A impact the broad association of RNA with chromatin, as well as cytological-scale chromatin condensation. We argue that seemingly contradictory effects of SAF-A loss and expression of various mutants are best understood in terms of dominant negative effects of mutant SAF-A that disrupts a complex RNP scaffold which supports nuclear chromosome architecture. Importantly, several results indicate the dominant negative effects are mediated by disruption of RNA’s relationship to chromatin.

### Brief review: the intersection of SAF-A, XIST RNA, and C_0_T-1 RNA

Early studies identified a number of proteins that remain with insoluble nuclear material after removal of most chromatin and nuclear proteins, giving rise to the debated concept of a non-chromatin nuclear “scaffold” or “matrix”, which was thought to underpin nuclear chromosome structure (reviewed in (Nickerson 2001)). One such protein thus identified was scaffold attachment factor A (SAF-A), so named because of its high affinity for certain sites on chromatin that resist nuclear extraction, suggested to be “scaffold attachment sites” (Fackelmayer et al. 1994). A ubiquitous, abundant, and highly conserved protein, SAF-A was independently identified as hnRNP U and implicated in RNA metabolism, including splicing (Kiledjian and Dreyfuss 1992). SAF-A is an essential protein, since loss of normal SAF-A is an embryonic lethal (Ye et al. 2015), and the interest in SAF-A is heightened further by recent studies that variants of SAF-A have been linked to several neurodevelopmental defects (Durkin et al. 2020).

**Fig. 1:**
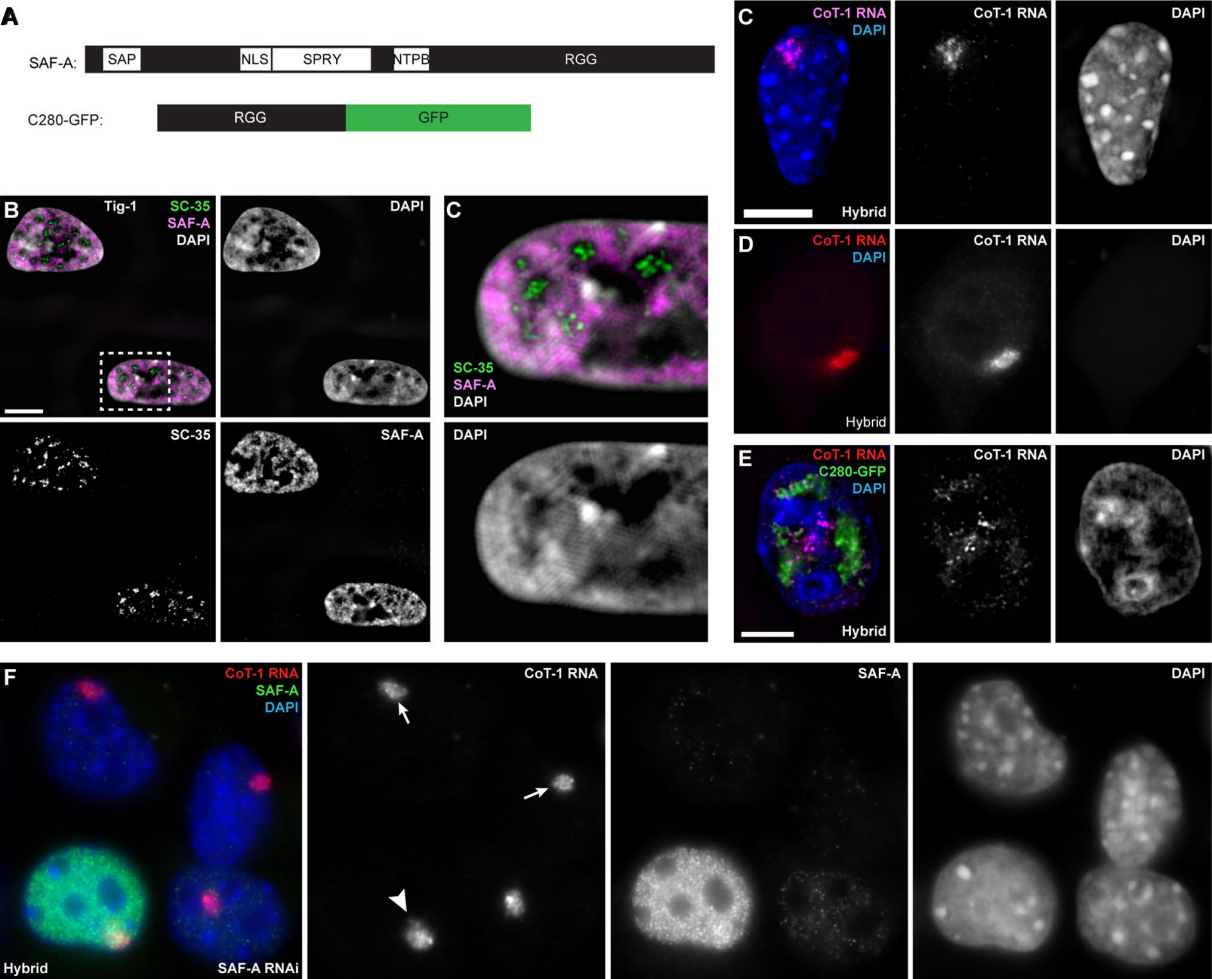
SAF-A localizes across chromatin and impacts Cot-1 RNA localization when mutated but not when depleted. For all images: color channels are separated in black and white. Scale bars 5 μm. Cell types: normal human fibroblasts (Tig-1) & mouse/human hybrid cells with human chromosome 4 (Hybrid). **A** Diagram of endogenous SAF-A protein and the human C280-GFP SAF-A deletion mutant. **B** SAF-A is detected over chromatin, but not within non-chromatin SC35 domains, as seen by structured illumination microscopy (SIM) image. **C** Close-up of indicated region of nucleus in image B (SIM). **D**–**E** Human C_0_T-1 RNA remains localized over the human chromosome in hybrid control cells (**D**) and in cells where DNA and histone chromatin proteins are removed (canonical nuclear matrix prep) (**E**). **F** Expression of the C280-GFP SAF-A mutant results in release of human C_0_T-1 RNA from the human chromosome in hybrid cells (SIM). **G** C_0_T-1 RNA remains localized to the human chromosome in hybrid cells when endogenous mouse SAF-A is present (short arrow) and when it is eliminated using siRNA (long arrows)

SAF-A contains multiple domains of interest, as summarized in Fig. 1A. SAF-A binds DNA, with high affinity for AT-rich scaffold or matrix attachment regions, through its N-terminal DNA binding SAP domain (Gohring and Fackelmayer 1997). An earlier in vitro study showed that SAF-A could cause DNA to organize into loops visible by electron microscopy (Fackelmayer et al. 1994), suggesting a potential role in chromatin packaging. More recently it was shown that SAF-A oligomerizes in vitro and in vivo, promoted by its binding RNA and hydrolyzing ATP (Nozawa et al. 2017). Thus SAF-A has been implicated to influence chromatin packaging at some level, as indicated by analysis of chromatin contacts assessed by high-throughput chromosome conformation capture (Hi-C) or pair-wise gene distances seen by fluorescence in situ hybridization (FISH) (Fan et al. 2018; Nozawa et al. 2017). Here our focus in on examining any effects of SAF-A on association of RNA with chromatin, but we also examine effects on cytological-scale chromatin condensation.

While the N-terminus has SAF-A’s DNA binding domain, SAF-A also has a C-terminal RGG box (Fig. 1A) which was shown to bind RNA in vitro (Kiledjian and Dreyfuss 1992; Thandapani et al. 2013). Subsequent studies have shown that SAF-A co-immunoprecipitates with a variety of RNAs, including many nuclear noncoding RNAs and mRNAs (Sharp et al. 2020; Xiao et al. 2012). Because of SAF-A’s separate DNA and RNA binding (RGG) domains, it provided a prime candidate for a protein that could act as a molecular bridge between RNA and DNA. As summarized below, initial findings of SAF-A’s effect on XIST RNA localization appeared to support this straightforward mechanistic role for SAF-A.

The clear precedent for a long RNA that embeds with the nuclear chromosome structure is XIST/Xist RNA (human and mouse X inactivation-specific transcript), which is essential for X-chromosome inactivation in mammalian female cells. This ~ 17 kb non-coding RNA is transcribed exclusively from the inactive X-chromosome where it accumulates and spreads to physically “paint” the chromosome territory in interphase nuclei (Brown et al. 1992; Clemson et al. 1996). XIST RNA induces cytological chromosome compaction, concomitant with modifications to histone and non-histone proteins, to stably silence the majority of X-linked genes (Brockdorff et al. 2020; Creamer and Lawrence 2017; Loda and Heard 2019). XIST is known to be required to initiate X-chromosome inactivation, however due to redundant layers of repressive factors, the heterochromatic chromosome remains essentially silenced if XIST RNA is experimentally lost from somatic cells. XIST RNA has mostly been studied for the numerous histone modifications the RNA triggers during the silencing process. However, the marked condensation of active-X chromosome architecture to form the heterochromatic structure called the Barr body (visible cytologically by DNA stains), may also involve changes to proteins of an architectural scaffold (including SAF-A).

Interactions between chromosomal RNAs and chromatin are increasingly recognized as essential for regulating the epigenome, with XIST RNA the pre-eminent paradigm. How XIST RNA interacts with chromatin and spreads across a whole chromosome is not well understood. Immunofluorescence to SAF-A is readily apparent across euchromatin, but antigen retrieval procedures that expose the SAF-A epitope in heterochromatin are necessary to reveal that it is broadly distributed across chromatin in general, including enrichment on heterochromatin of the inactive X-chromosome (Xi). This enrichment of SAF-A on the Xi first linked SAF-A to XIST RNA (Helbig and Fackelmayer 2003), and this coupled with the protein’s ability to bind both DNA and RNA by separate domains, raised interest in whether this abundant scaffold protein had a role in XIST RNA localization. From the initial study in mouse cells it appeared that SAF-A/hnRNP U acts as a unimolecular bridge that is both necessary and sufficient to tether Xist RNA to Xi chromatin (Hasegawa et al. 2010), and this became the prevailing view (Gendrel and Heard 2014; Nakagawa and Prasanth 2011). However, our lab subsequently examined this in a number of human (and mouse) cell types and found conflicting evidence in normal cells versus certain tumor cells, indicating more biological complexity (Kolpa et al. 2016). Through a series of experiments using siRNA to deplete SAF-A in different cell types, Kolpa et al. found that robust depletion of SAF-A had no effect on XIST/Xist RNA localization in normal human or mouse cells. However, consistent with Hasegawa et al., Kolpa et al. found SAF-A RNAi completely released Xist RNA in the mouse tumor cell line (Neuro2a). Hence, Kolpa et al. suggested that SAF-A is one of multiple proteins in the nuclear scaffold that support XIST RNA localization, providing functional redundancy in normal cells, but during transformation loss of one or more proteins that are functionally redundant to SAF-A can compromise RNA-anchoring in some cancer cells (best illustrated by Neuro2a tumor cells). Although loss of SAF-A by RNAi had no or negligible effect on XIST RNA in normal cells, a SAF-A deletion mutant lacking the DNA binding domain did disrupt XIST RNA’s localization to the Xi chromosome.

In sum, these findings supported that SAF-A has some relationship to XIST RNA association with the inactive X-chromosome, but also indicated these interactions are more complex than a single-protein that is necessary and sufficient to bridge XIST RNA to DNA. Results also made clear that the factors impacting XIST RNA localization can differ between normal and transformed cells. Most relevant to the experimental work presented here, the lack of effect of SAF-A RNAi on XIST RNA, compared to pronounced effect of a SAF-A DNA binding mutant, appeared contradictory; however, as explained below, the results are consistent with other evidence presented here that SAF-A mutants can have dominant negative effects which depletion of SAF-A does not.

In contrast to XIST RNA which produces and maintains heterochromatin, long repeat-rich RNAs, detected by hybridization with a probe to C_0_T-1 DNA (highly repetitive genomic DNA) are abundant across euchromatin, and absent on the inactive X-chromosome and peripheral heterochromatin (Hall et al. 2014). As most clearly shown by RNA FISH in hybrid cells carrying a single human chromosome, human C_0_T-1 RNA localizes to the parent chromosome territory. A recent study from our lab (Creamer et al. 2021) developed a procedure to selectively separate most (~ 85%) nuclear RNAs from RNAs that co-fractionate with XIST by removal of weakly bound RNAs and proteins (with urea) followed by salt extraction of histones and other nuclear proteins and digestion of DNA to undetectable levels. Remarkably, XIST RNA remains in a bright localized RNA territory, on a non-chromatin “scaffold” of the nuclear chromosome territory. Other highly insoluble RNAs that co-fractionate with XIST RNA in the non-chromatin scaffold were isolated and sequenced. This scaffold RNA is collectively detected with a C_0_T-1 DNA probe, as it was found to comprise overwhelmingly non-coding and repeat-rich sequences within long, largely nascent transcripts (Creamer et al. 2021). Creamer et al. further found that in human fibroblasts in which mRNA synthesis was inhibited by DRB, there was extensive production of long, new intergenic, or down-stream transcripts that essentially maintained the mass of scaffold RNA. Importantly, inhibition conditions that maintain the C_0_T-1 RNA on the chromosome territory also maintain normal cytological-scale chromatin distribution and vice versa.

Creamer et al. (2021) further manipulated scaffold RNA abundance or localization by depletion with RNase, transcriptional arrest, or by physical disruption. In each case a strong inverse correlation between the distribution of C_0_T-1 RNA and the distribution of condensed chromatin was observed. A similar relationship was found between specific nuclear scaffold proteins that co-distribute on euchromatin with C_0_T-1 RNA, including SAF-A. Collectively, results indicated that long, nascent transcripts platform dynamic, but structurally robust insoluble RNP structures that physically antagonize chromatin compaction.

Since SAF-A has been reported to interact with specific lncRNAs as well as mRNAs (Hacisuleyman et al. 2014; Puvvula et al. 2014; Sharp et al. 2020), how loss or mutation of SAF-A impacts the relationship of these RNAs to chromatin is of substantial interest, as investigated here. Our findings point to much more complex effects of SAF-A perturbations than is compatible with the view of SAF-A as a unimolecular bridge between RNA and chromatin. Rather, results provide further evidence for a model whereby SAF-A is one of numerous RNA binding proteins in an insoluble network platformed by long, chromatin-associated RNAs, referred to here as the “RNP scaffold”.

Here we present a series of experiments manipulating SAF-A to examine the effects on C_0_T-1 hnRNA’s broad association with chromatin and show evidence that SAF-A is not a “tether” between RNA and DNA, but rather, SAF-A mutants influence a more complex RNP scaffold that may in turn impact chromatin condensation.

## Results

### SAF-A like C_0_T-1 localizes primarily to interphase chromatin and is released at mitosis

First, we consider the distribution of SAF-A in nuclear structure which gives some insight into its role. As summarized above, SAF-A was isolated as an abundant component of the operationally defined nuclear scaffold (Fackelmayer et al. 1994), but was also originally classified as an hnRNP protein and believed to have a role in pre-mRNA splicing (Kiledjian and Dreyfuss 1992). SAF-A was pulled down as part of a major splicing complex (C complex) (Jurica et al. 2002), and purified from mouse interchromatin granule clusters (also known as Speckles or SC-35 domains) (Saitoh et al. 2004), which are non-chromatin domains of concentrated pre-mRNA splicing factors (Hall et al. 2006; Spector and Lamond 2011), although a subsequent study did not find SAF-A is part of the spliceosome (Zhou et al. 2002). More recent studies showed immunofluorescence (IF) for SAF-A distributes broadly across euchromatin but appeared not to localize with heterochromatin (Nozawa et al. 2017; Sunwoo et al. 2017), despite prior evidence of its enrichment on Xi. To better clarify SAF-A distribution we used procedures to maximize detection of the epitope by IF (antigen retrieval), and used high-resolution structured illumination microscopy (SIM). Results showed SAF-A is largely excluded from SC-35 speckles, as is DNA (Carter et al. 1991), and instead localized over DNA (Fig. 1B, C, Suppl Fig. 1A). We affirm that it localizes to both euchromatin and heterochromatin, but this requires antigen retrieval or nuclear matrix procedures that increase detection of the epitope in heterochromatin, particularly over the Barr body (Xi) (Suppl Fig. 1B, C) (Helbig and Fackelmayer, 2003; Kolpa et al., 2016).

SAF-A is released from chromosomes in early prophase of mitosis, as is XIST RNA, C_0_T-1 RNAs, and several other non-chromatin nuclear structural proteins (Suppl Fig. 1A & D). Hence, during the cell-cycle, changes in the chromatin association of both SAF-A and RNA roughly coincide, detaching during mitosis, and reappearing on chromatin in early G1.

### A highly truncated SAF-A protein releases C_0_T-1 RNA from chromatin, whereas SAF-A depletion does not

Because SAF-A contains both DNA and RNA binding domains, it was proposed to act as a molecular bridge between RNA and chromatin. We previously showed that repeat-rich heterogenous nuclear RNA (hnRNA), detected using a C_0_T-1 DNA probe, strictly localizes to its parent chromosome territory in interphase, and resists extraction by classical nuclear matrix isolation procedures (Fig. 1D, E) (Hall et al. 2014). We recently developed an improved protocol that thoroughly removes DNA and ~ 85% of nuclear RNA, and C_0_T-1 RNA remains undisturbed with the insoluble territory “scaffold” (Creamer et al. 2021). While SAF-A was not studied in detail, we initially showed the C_0_T-1 RNA can be released to disperse by expression of a truncated SAF-A mutant (C280-GFP), which lacks all but the C-terminal 280 amino acids and still contains the RGG which binds RNA (Fig. 1A). Thus, transient expression of this mutant shows C_0_T-1 RNA can be released from chromosome structure, and dispersed RNA is not rapidly degraded (Hall et al. 2014) (Fig. 1F). While this implicated SAF-A to play some role in C_0_T-1 hnRNA’s association with chromatin, we emphasized that it was not clear whether SAF-A is required to directly tether C_0_T-1 hnRNA, or whether the C280-GFP mutant could have indirectly disrupted an RNA localization by disrupting a putative chromosomal scaffold.

To investigate whether SAF-A is required to maintain C_0_T-1 RNA localization, we used siRNA to deplete SAF-A in mouse–human hybrid cells for 72 h. Use of hybrid cells was needed for clear visualization of whether human C_0_T-1 RNA remained localized on the single human chromosome. Previously, we showed highly effective knock-down of SAF-A by RNAi in other cell types (Kolpa et al. 2016); however, we quantified that again for these hybrid cells, using a single-cell assay that avoids effects of transfection efficiency. Whereas SAF-A staining is normally bright in all cells, the robust knock-down of SAF-A by siRNA is evident in Fig. 1G; we also affirmed by microfluorimetry on many individual cells that SAF-A depletion was highly effective, reducing SAF-A levels in transfected nuclei by 97% compared to neighboring untransfected cells. Only these cells lacking visible SAF-A staining were scored for effects of SAF-A knock-down (cells with weak SAF-A staining were not included) non-transfected cells on the same slide (with normal SAF-A staining) served as internal controls. Surprisingly, in marked contrast to the impact of the truncated protein (C280-GFP), SAF-A depletion had no visible effect on the C_0_T-1 RNA territory in all transfected cells (nor on overall DNA distribution, further discussed below) (Fig. 1G). These experiments were repeated three or more times with reproducible results. We also tested in multiple experiments two other human cell lines: primary Tig-1 fibroblasts and transformed Hek 293 cells, where again C_0_T-1 RNA and cytological chromatin distribution both appeared unaffected by SAF-A depletion (Suppl Fig. 1E, F). These results indicated that the localization of C_0_T-1 hnRNA to chromatin was not directly dependent upon tethering by SAF-A, as had been proposed and debated for XIST RNA (summarized above).

The fact that the C280 mutant has a much greater effect on XIST (Hall et al. 2014; Kolpa et al. 2016) and C_0_T-1 RNA localization than SAF-A depletion provided the first indication that the mutant acts more indirectly via a dominant negative effect on some other factor. As further considered below regarding analysis of other mutants, there is some heterogeneity of SAF-A mutant effects within a cell population, particularly in transformed cell lines which have inherent variability, but this can also be impacted by expression levels, or, as explained below, whether a cell divided in the presence of the mutant. Control comparisons with GFP-tagged wild-type SAF-A indicated that cells with very high expression of even wild-type (GFP-tagged) SAF-A caused aberrant nuclear/chromatin structure in a subset (~ 20%) of cells; therefore, throughout these experiments analysis of results excluded cells with extremely bright GFP (protein), as well as very weakly expressing cells. This became most important for quantification of results on other mutants below for which more heterogeneity was observed. While there was some variability in the patterns observed with the C280 mutant expression, this highly truncated SAF-A consistently had obvious effects on nuclear chromatin structure and RNA distribution, further suggesting likely dominant negative effects on nuclear structure.

**Fig. 2:**
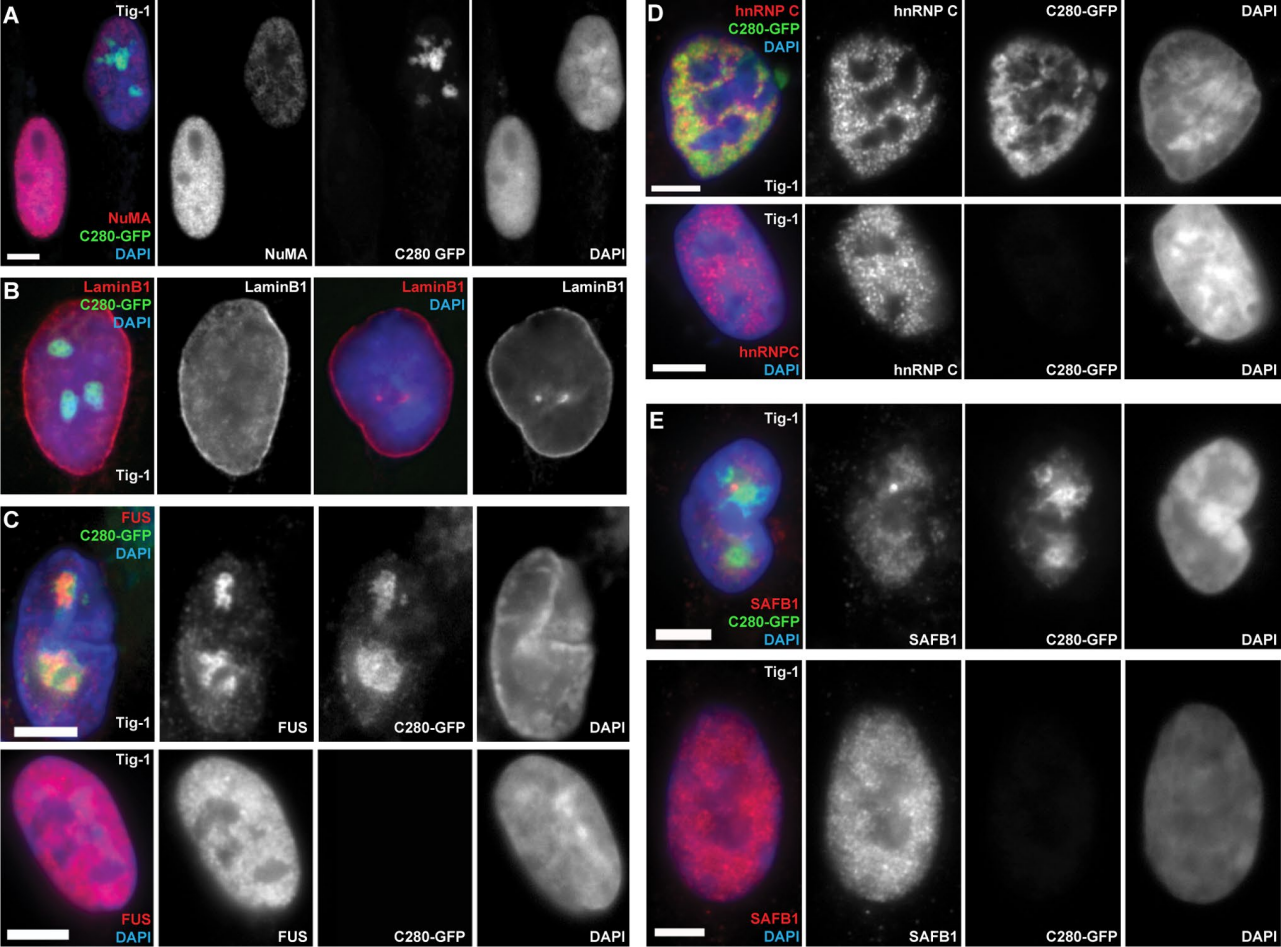
C280 SAF-A mutant displaces specific nuclear proteins and not others. For all images: color channels are separated in black and white. Scale bars 5 μm. Cell types: normal interphase human fibroblasts (Tig-1). **A**–**B** Expression of the C280-GFP SAF-A mutant had no effect on the overall nuclear distribution of LaminB1 (**B**) or NuMA (**A**) (top cell was transfected with the C280 vector while bottom cell was not). However, the deletion mutant frequently led to reduced levels of NuMA. Notably, in many cells the deletion mutant disrupts the normal distribution of FUS (**C**), hnRNPc (**D**), and SAFB1 (**E**). All nuclei shown are in interphase (not prophase or mitosis) and changes in DNA corresponded to presence of SAF-A mutant. Control cells lack green C280-GFP signal. Additional images in Supplemental Fig. 2 and 3

We therefore surveyed whether expression of the C280 SAF-A mutant leads to perturbation of other important nuclear structural proteins. Creamer et al. recently revealed that in normal cells, IF for SAF-A as well as other well-known nuclear matrix proteins, NuMA and Matrin-3, co-distribute with C_0_T-1 RNA specifically in euchromatin (whereas Lamin A/C distributes more broadly). Hence, it was of interest to determine if perturbing SAF-A would impact other nuclear structural proteins. Here we examined C280 SAF-A mutant effects on NuMA, Lamin B1, SAFB1, FUS, and hnRNP C. Figure 2 shows representative examples of the effects seen on distribution of these different proteins, including examples of some inter-cellular variation seen for certain proteins. As illustrated in the examples shown (Fig. 2 & Supplemental Fig. 2), this truncated SAF-A had no discernible impact on distribution of NuMA or Lamin B1 (Fig. 2A, B & Supplemental Fig. 2A, B). However, the SAF-A C280 deletion mutant led to reduced levels of NuMA and in many cells grossly disrupted distributions of hnRNP C and FUS (Fig. 2C, D & Supplemental Fig. 2C, D). While the patterns of localization varied between cells in a population, in sub-set of cells, FUS specifically was found to co-localize with C280-GFP aggregates (Fig. 2C), suggesting that FUS may interact more directly with the mutant SAF-A protein.

These results show that the mutant C280 SAF-A impacts certain other major structural proteins, bolstering the evidence that the effects of the C280 mutant protein are likely indirect and involve broader perturbation of other factors that interact with C_0_T-1 RNAs.

### A single-point mutation in the DNA binding domain of SAF-A mis-localizes C_0_T-1 RNA from chromatin

**Fig. 3:**
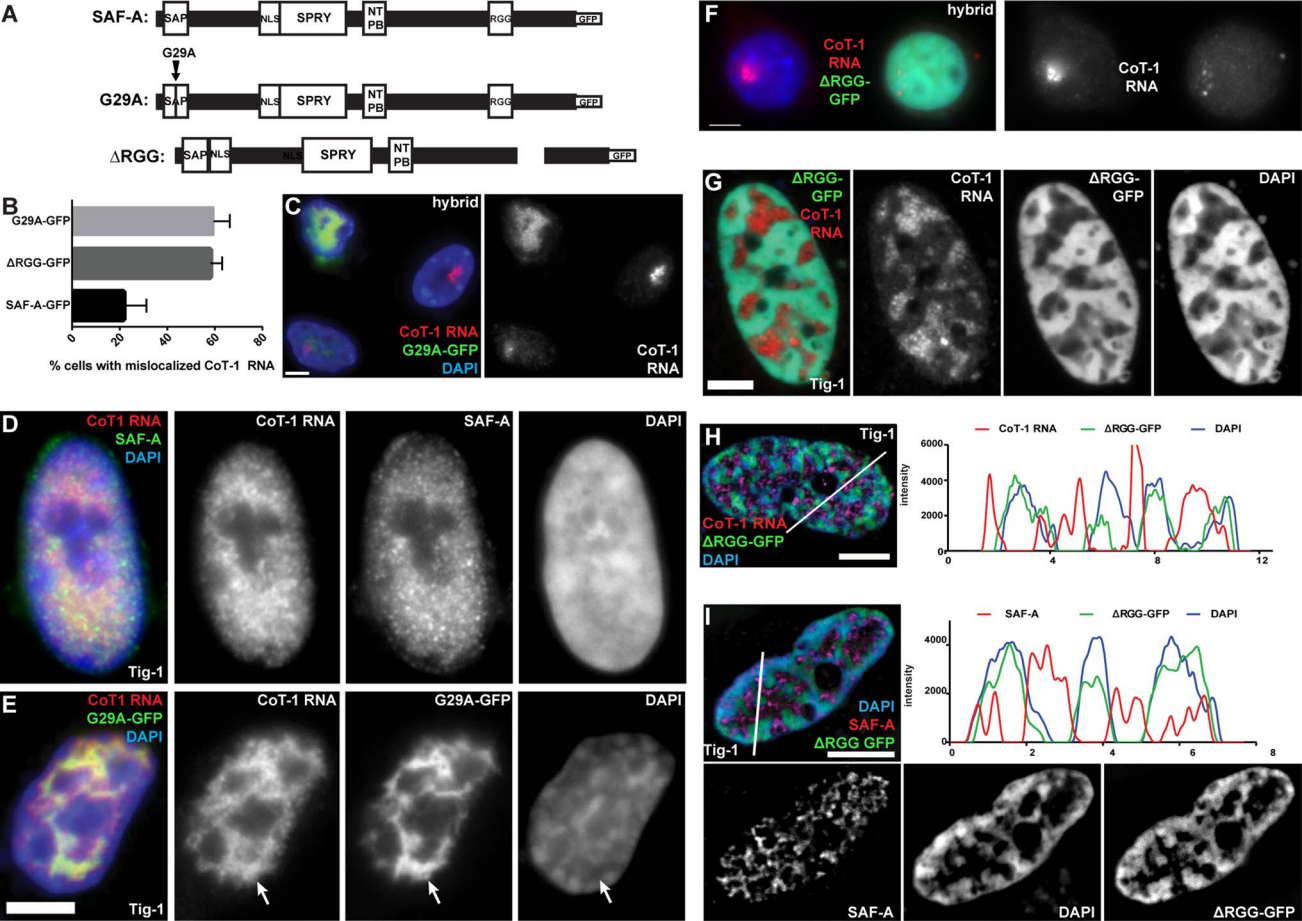
Both SAF-A DNA binding and RGG binding domain mutants displace C_0_T-1 RNA and alters DNA morphology. For all images: color channels are separated in black and white. Scale bars 5 μm. Cell types: mouse/human hybrid cells with human chromosome 4 (Hybrid) & normal human fibroblasts (Tig-1). **A** Map of full-length SAF-A, and the G29A and ∆RGG mutants. **B** Both SAF-A mutants affect C_0_T-1 RNA localization to chromatin (G29A: *P* = 0.001, RGG: *P* = 0.004). Error bars, standard deviation of the mean. Wildtype SAF-A-GFP also affected C_0_T-1 RNA localization when it was grossly overexpressed (in ~ 20% of cells). **C** Expression of the G29A mutant releases C_0_T-1 RNA from the human chromosome in hybrid cells, while non-transfected neighboring cells are unaffected. **D**–**E** Normal C_0_T-1 RNA distribution in fibroblasts (**D**) is altered when the G29A mutant is expressed (**E**). C_0_T-1 RNA localizes with G29A-GFP and not with DAPI DNA staining (arrows), and DNA condensation is altered. **F** Expression of the ∆RGG mutant mis-localizes human C_0_T-1 RNA from the human chromosome in hybrid cells compared to a neighboring cell that was not transfected. **G** C_0_T-1 RNA is released from chromatin in fibroblasts expressing the ∆RGG SAF-A mutant, and DNA compaction is altered. **H** SIM image and linescan showing relative distributions and intensity of DAPI DNA, C_0_T-1 RNA, and ∆RGG SAF-A mutant in a fibroblast nucleus. **I** SIM image and linescan showing distribution of DAPI DNA, endogenous SAF-A and ∆RGG SAF-A mutant in a fibroblast nucleus

To better understand the interplay of SAF-A on localization of RNA to chromatin, we examined the effects on C_0_T-1 RNA localization using defined mutations of SAF-A’s DNA and RNA binding domains. While the C280 mutation deleted multiple domains of SAF-A (Fig. 1A), we examined a DNA binding domain mutant (G29A-GFP) which has a single nucleotide substitution in the DNA binding domain and is tagged with GFP for visualization (Fig. 3A). Remarkably, in multiple experiments with scoring hundreds of cells, this precise mutation (G29A) results in release of C_0_T-1 RNA from the chromosome territory in most hybrid cells expressing it (~ 60% *P* = 0.0016), with greater effect in cells with higher expression (Fig. 3B, C). We repeated this experiment in normal Tig-1 fibroblasts, where visualization of C_0_T-1 release is somewhat more difficult due to C_0_T-1 RNAs broad and uninform distribution across euchromatin. However, C_0_T-1 RNA no longer uniformly distributed on chromatin in 82% of cells expressing the G29A mutation (as noted above, scoring eliminated cells with marked overexpression of G29A, see Methods). This confirmed the marked mis-localization of RNA that was clearly evidenced in the hybrid cells. Interestingly, G29A-GFP protein distribution showed a clear pattern of concentration in DAPI-depleted nuclear regions, and, furthermore, C_0_T-1 RNA colocalized with mutant SAF-A in these regions (Fig. 3D, E & Suppl Fig. 3A–C). Thus, the G29A SAF-A mutant, which can no longer bind chromatin (through its DNA binding domain) can still interact with C_0_T-1 RNA (through its functional RGG domain), appearing to sequester C_0_T-1 RNA away from chromatin in DNA depleted regions.

These results may initially appear to suggest that SAF-A’s ability to bind DNA is required for C_0_T-1 RNA’s localization to chromatin, however, further scrutiny suggests an alternative explanation. As illustrated in Fig. 3 (Fig. 3A, E and Suppl Fig. 3A, B), C_0_T-1 RNAs co-localize with the G29A-SAF-A protein and very likely still bind the mutant SAF-A’s RGG domain. However, that protein no longer binds DNA. Hence, it may be SAF-A’s ability to bind RNA, in the absence of its capacity to bind DNA, that impacts C_0_T-1 RNA’s chromatin association. Unbound SAF-A that contains a functioning RGG domain causes displacement of C_0_T-1 RNA, in effect “stripping” it from chromatin. This finding is key to understanding why in our experiments the robust depletion of SAF-A (via RNAi), to often cytologically undetectable levels, did not discernibly mis-localize C_0_T-1 RNA, whereas the presence of this SAF-A mutant does. That this effect is mediated by effects on RNA is further suggested by results below.

### SAF-A mutant lacking the RGG domain causes displacement of C_0_T-1 RNAs and endogenous SAF-A from chromatin

We next examined the effects on C_0_T-1 RNA localization of a mutant SAF-A lacking the RGG domain but retaining the DNA binding domain (ΔRGG-GFP) (Fig. 3A). Our SAF-A RNAi results suggest that SAF-A is not required for C_0_T-1 RNA localization, and the above results suggest the unbound G29A mutant displaces RNA from chromatin because it retains the RGG domain. This might then predict that a SAF-A mutant lacking the RGG domain may not mis-localize RNA from chromatin. However, we found that C_0_T-1 RNA again became mis-localized off chromatin in over half of hybrid cells expressing ΔRGG-GFP (58% *P* = 0.004) (Fig. 3F & Supplemental Fig. 3D) (again eliminating highly expressing cells). Similar effects on C_0_T-1 RNA localization are seen in normal human Tig-1 fibroblasts expressing the ΔRGG-GFP mutant for 24 h. The somewhat diminished C_0_T-1 RNA signal clearly did not localize with the ΔRGG mutant SAF-A (Fig. 3G, H), supporting that the RGG domain is required for RNA binding. In fact, C_0_T-1 RNA signal was restricted to DAPI-depleted areas in 53% of cells, while the ΔRGG SAF-A mutant protein, with a functional DNA binding domain, remained localized to chromatin.

Given that the above siRNA results found SAF-A was not necessary for C_0_T-1 RNA to localize to chromatin, the question becomes: why does the ﻿ΔRGG SAF-A impact C_0_T-1 RNA-chromatin association, even in cells that still contain endogenous SAF-A? To address this, we examined localization of endogenous SAF-A with an antibody that does not recognize the ∆RGG mutant. Strikingly, this revealed that in 96% of ∆RGG-GFP-positive cells with abnormal C_0_T-1 RNA distribution, the majority of endogenous SAF-A no longer associates with chromatin but co-localizes with released C_0_T-1 RNA in “DAPI holes” lacking substantial DNA (Fig. 3I). Thus, both C_0_T-1 RNA and normal SAF-A with its functional RGG domain now mis-localize together off chromatin, whereas the ΔRGG mutant protein has largely displaced endogenous SAF-A on chromatin.

Hence, while our quantification showed heterogeneity in the mutant’s effects within the cell population, this key finding proved highly consistent: essentially all cells with displaced C_0_T-1 RNA show similar displacement of endogenous SAF-A from chromatin. Therefore, rather than concluding that SAF-A’s RGG domain is required to maintain C_0_T-1 RNA on chromatin, these results reveal that the ∆RGG-GFP mutant has a dominant negative effect by displacing endogenous SAF-A from chromatin, which in turn causes displacement of C_0_T-1 RNA. This has parallels to the dominant negative effect seen with the DNA binding mutant, where mis-localized SAF-A molecules with functioning RGG domains disrupt RNAs from chromatin, whereas loss of wild-type SAF-A did not.

### Endogenous SAF-A requires RNA to bind chromatin but mitotic release of SAF-A is impeded by GFP-tag

**Fig. 4:**
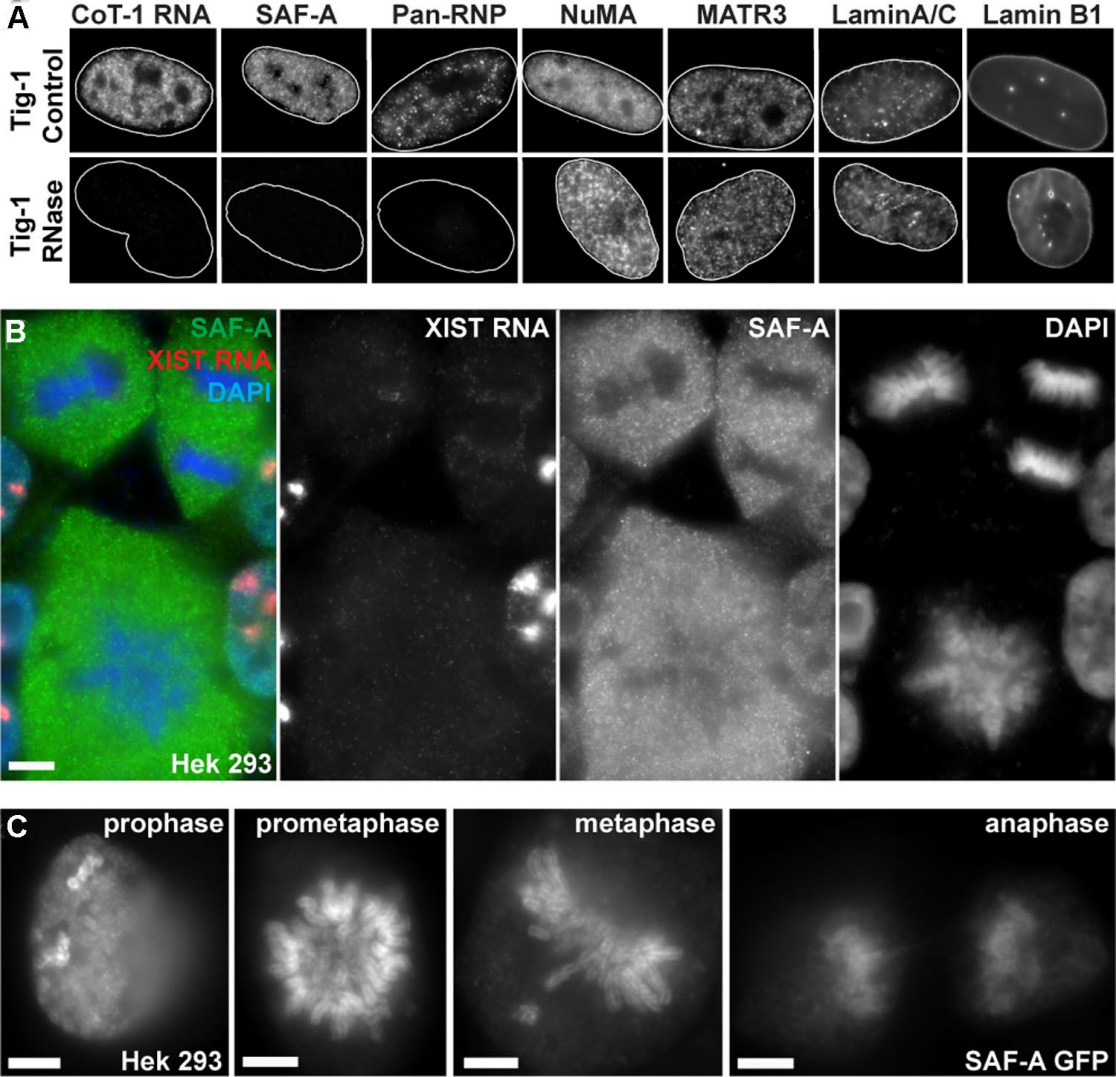
RNA-dependent and independent nuclear protein structure and effects of GFP-tags on SAF-A binding. For all images: scale bars 5 μm. Cell types: normal human fibroblasts (Tig-1) & immortalized human female kidney cells (﻿Hek293). **A** SAF-A and Pan-RNP proteins are released when C_0_T-1 RNA is digested with RNase in normal fibroblast cells, while NuMa, MATR3, Lamin A/C, and Lamin B1 are not. Outline of nuclei (delineated by DAPI DNA) in white for all but LaminB1. **B** Endogenous full-length SAF-A releases from chromosomes during normal mitosis in Hek293 cells. Color channels are separated to the right. **C** In contrast, when tagged with GFP, the normal full-length SAF-A protein remains bound to mitotic chromosomes throughout mitosis. Note enrichment of SAF-A on both inactive X-chromosomes in Hek293 prophase cell

It is a curious finding that the mutant SAF-A lacking the RGG is bound to chromatin and even appears to “out-compete” endogenous SAF-A for chromatin localization. All above results indicate that the RGG domain is required for SAF-A to bind RNA, and other evidence indicates SAF-A requires RNA to bind to chromatin. RNase treatment leads to loss of SAF-A from nuclei (Nozawa et al. 2017), but since RNase treatment will have broad and potentially indirect effects, it is instructive to examine the most immediate effects of RNase on SAF-A in comparison to several other nuclear structural proteins. In Creamer et al. (2021) we recently showed that in just one minute of RNase, SAF-A is completely lost from permeabilized nuclei, whereas Lamin B1, NuMA, and Matrin-3 were retained after 10 min. Here we expand on this by examining other nuclear scaffold proteins after treating unfixed, permeabilized Tig-1 fibroblasts with RNase. IF for SAF-A confirms SAF-A quickly disappears (within 1–5 min at 37 °C) (Fig. 4A), and we similarly examined Lamins A/C, Lamin B1, NuMA, and a Pan-hnRNP antibody (which broadly detects hnRNP proteins). While SAF-A and Pan-hnRNP are eliminated by RNase, NuMA, and both lamins were retained (Fig. 4A). Hence, this suggests there are RNA-dependent and independent “layers” in nuclear chromosome structure, and SAF-A is among the RNA-dependent factors that requires RNA to bind chromatin.

Other observations provide insight into the apparent contradiction that the RGG mutant still binds chromatin and also provide important information on the potential impacts of commonly used protein tags. SAF-A has a long half-life on chromatin, so the mutant protein will likely gain access to compete with the wild-type protein when endogenous SAF-A releases from chromatin during mitosis. We considered that whether or not a cell has divided during the period after transfection might account for the incomplete penetrance of SAF-A mutant effects on C_0_T-1 RNA (impacting ~ 50–60% of transfected cells). Importantly, in examining mitotic cells we discovered that the GFP-tag causes abnormal binding of SAF-A throughout mitosis (of both full-length and mutant SAF-A). As shown in Fig. 4B, endogenous SAF-A is normally dispersed to the cytoplasm at the onset of mitosis when C_0_T-1 hnRNA is released (Hall et al. 2014). However, we discovered that the GFP-tag causes even the normal full-length SAF-A to remain bound throughout all of mitosis (Fig. 4C). SAF-A is known to undergo multiple phosphorylation events, with which the GFP-tag likely interferes. The ∆RGG-GFP mutant still has the DNA binding domain, and this demonstrated effect of the GFP-tag that blocks release of normal SAF-A at mitosis explains why the RGG mutant with GFP-tag does not release at mitosis, and therefore out-competes chromatin binding of endogenous SAF-A.

This finding not only explains the effects of this SAF-A mutant, but awareness of the demonstrated effect of the GFP-tag is important and should be considered for potential effects on other proteins. Other details regarding effects of GFP or Flag tags are provided in Methods, however the central point here is that all three SAF-A mutants disrupt RNA association with chromatin, not because normal SAF-A is required, but because mutant SAF-A was disruptive to RNA’s interaction with chromatin.

### SAF-A mutants that disrupt euchromatin-associated RNA cause chromatin condensation

As shown above, all three mutants disrupt the normal chromatin distribution of abundant, heterogenous, and repeat-rich nascent RNAs. As summarized above, Creamer et al. (2021) recently used numerous other approaches to perturb RNA localization across euchromatin, which consistently caused rapid DNA condensation; this study further showed that long nascent RNAs serve as a platform for insoluble RNP structures that contribute to chromosome architecture. Here, the SAF-A mutants provide an alternative means to disrupt RNA-chromatin interactions and alter chromatin distribution. In several figures above it is evident (from the DAPI DNA staining) that the SAF-A mutants not only disrupt C_0_T-1 RNA localization, but also cause aberrant chromatin packaging on a cytological scale (e.g., Figures 1F, 2, 3E, G–I).

**Fig. 5:**
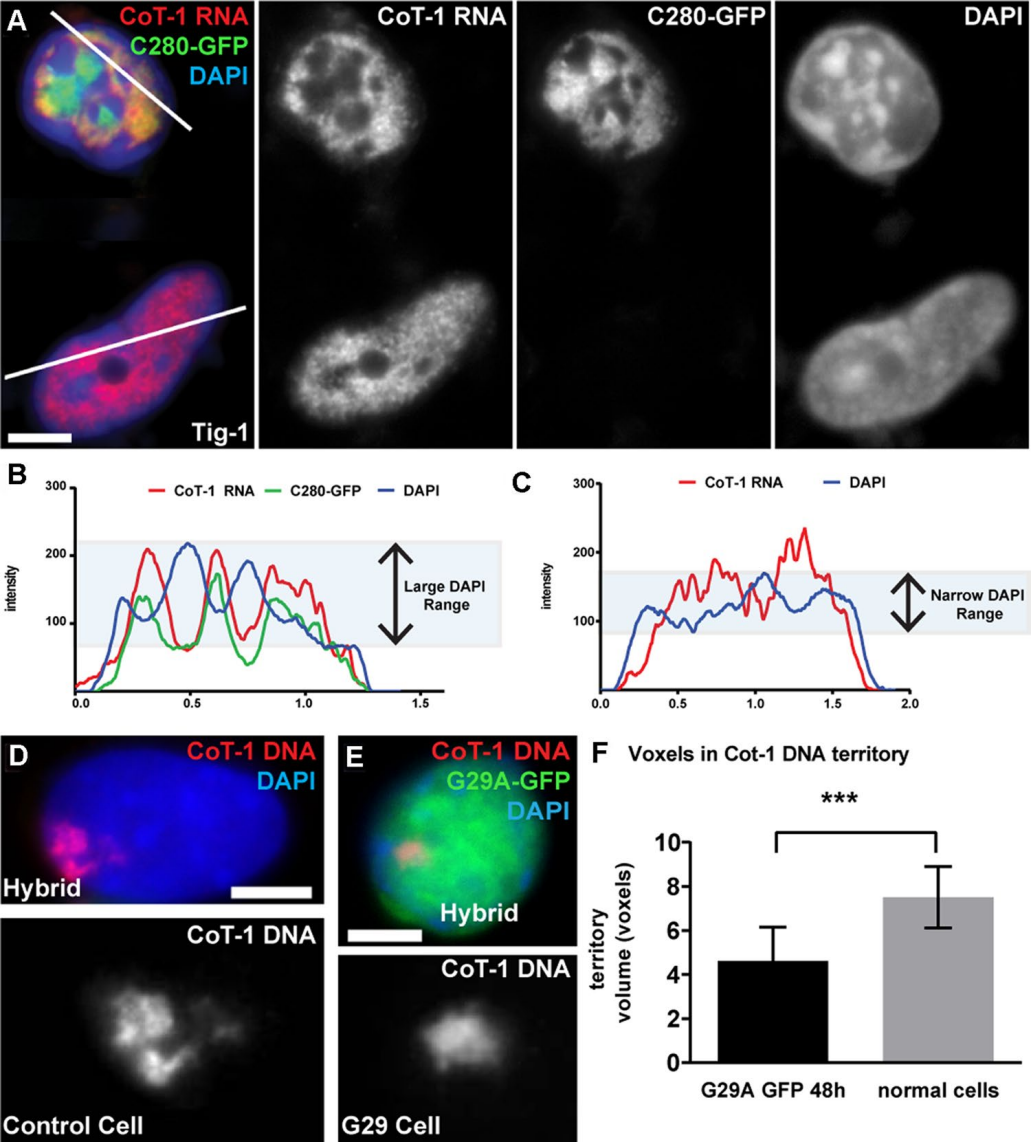
SAF-A mutants that release C_0_T-1 RNA affect cytological chromatin packaging. For all images: color channels are separated in black and white. Scale bars 5 μm. Cell types: normal human fibroblasts (Tig-1) & mouse/human hybrid cells with human chromosome 4 (Hybrid). **A** C280-GFP expression causes C_0_T-1 RNA to release and DAPI to condense compared to un-transfected neighboring cell. **B**–**C** Linescan of C280 expressing cell (**B**) and untransfected neighboring cell (**C**) showing larger variation in DAPI intensity measurements for C280 positive cell. C_0_T-1 RNA localizes with C280 and not with DAPI. Linescan paths indicated in A). **D**–**F** The human Chr4 territory (identified by human C_0_T-1 DNA) is larger in control hybrid cells (**D**) and condenses in hybrid cells expressing the G29A mutant (**E**), as quantified in (**F**). **F** The total volume of the human chromosome territory decreases by 38% in G29A expressing cells (*P* = 0.0002, *n* = 11). Error bars, standard deviation of the mean. Z-stacks deconvolved and voxel volume measured using Huygens software

We further characterized and quantified effects of the C280 and G29A SAF-A mutants on the cytological distribution of DNA, and in relation to localization of RNA and the mutant proteins. Based on analysis of hundreds of cells in multiple independent experiments, we found that ~ 50% of C280-GFP positive human cells contained large clumps of chromatin, indicated by larger peaks and valleys in DNA staining intensities compared to more uniform staining in control cells (Fig. 5A–C). Moreover, these DNA clumps lacked both C280-GFP protein and C_0_T-1 RNA, which co-localize in DNA depleted areas in 71% of cells with collapsed chromatin. This supports that unbound SAF-A mutants like C280, with functioning RGG domains, cause displacement of C_0_T-1 RNA from chromatin. This further supports other evidence that stripping RNA from chromatin causes it to condense, and that Cot-1 RNAs promote open chromatin structure through their interaction with a network of insoluble scaffold factors, one of which is SAF-A (Creamer et al. 2021).

Additionally, DAPI staining showed similar abnormal clumps of DNA in 82% of cells expressing the other DNA binding mutant (G29A-GFP) (Fig. 3D, E). As in other experiments above, we excluded from quantitation those cells with very highly expressed GFP mutant, given that in cultures transfected with wild-type GFP-SAF-A there is a subset of over-expressing cells with impact on RNA distribution with chromatin (see Fig. 3B), which in turn can cause chromatin aberrations (unrelated to the mutation in SAF-A). To more thoroughly examine chromatin condensation caused by this mutant, we measured its impact on the size of the single human chromosome (Chr 4) DNA territory in mouse hybrid cells expressing G29A-GFP; the human chromosome is delineated by hybridization with the human C_0_T-1 DNA probe (which does not detect mouse repetitive DNA) (Fig. 5D–F). As measured in deconvolved z-stacks (see Methods), the total volume of the human chromosomal DNA territory decreased in cells expressing the mutant SAF-A, as compared to untreated control cells. This provides direct quantitative evidence for contraction of an active chromosome territory following C_0_T-1 RNA loss.

With the SAF-A mutants that no longer bind DNA, a significant question is whether the aberrant condensation of chromatin is due to loss of RNA or loss of SAF-A from chromatin. Importantly, however, chromatin also condenses in most cells expressing ΔRGG-GFP, in which the mutated SAF-A remains with chromatin, but C_0_T-1 RNA has been displaced (Fig. 3G–I). This suggests that the often-dramatic impact on overall chromatin condensation seen for the DNA binding mutants (C280 and G29A) is not primarily due to the loss of SAF-A, but to the release of RNA from chromatin. In further support of this, in cells examined here, siRNA depletion of endogenous SAF-A neither releases C_0_T-1 RNA nor did it have cytologically discernible impact on chromatin distribution (Fig. 1G & Supplemental Fig. 2E, F). We do not rule out more subtle effects of SAF-A RNAi in certain cell lines, but clearly the impact of SAF-A mutants is far more marked and dramatic than any effect of SAF-A RNAi.

In sum, lack of C_0_T-1 RNA, and not lack of SAF-A, is the one condition that was consistently associated with obvious cytological chromatin condensation in all conditions.

## Discussion

**Fig. 6:**
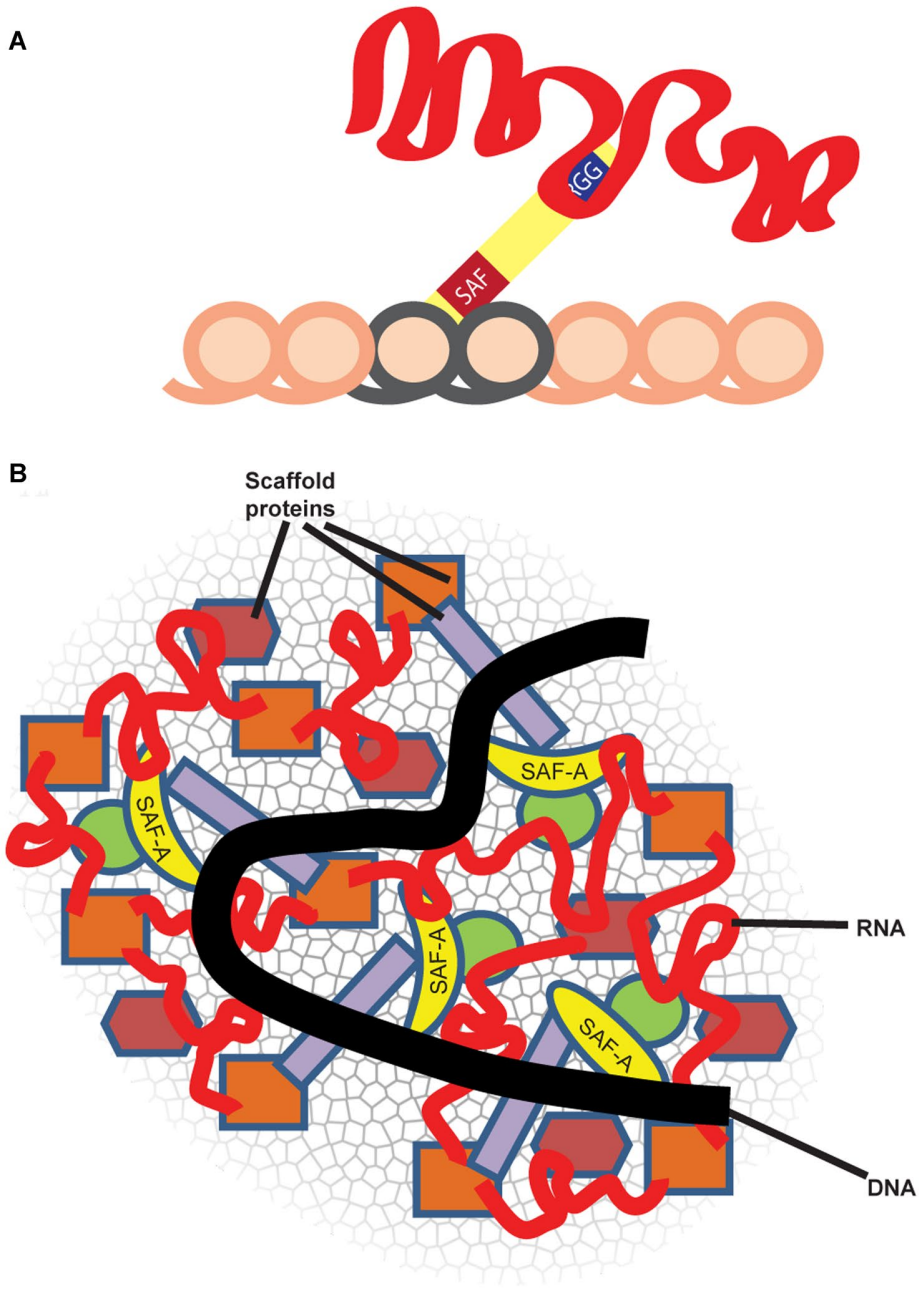
Two contrasting models of RNA localization to chromatin and role of SAF-A: **A** RNA tethered to chromatin via SAF-A or related anchors: initial studies suggesting SAF-A was required to localize XIST RNA appear to support a model whereby SAF-A acts as a single-molecule bridge between RNA and chromatin, through its separate RNA and DNA binding domains. In this model, the RNA impacts chromatin by recruiting histone modifiers, as XIST RNA is known to do. **B** RNA is a platform for an RNP network, including SAF-A, that physically impacts chromosome structure. In this, the “Its complicated” model, SAF-A is one component that associates with chromatin in an RNA-dependent manner, and long RNAs and SAF-A are woven into a “fabric” of the interphase chromosome, with SAF-A one (important component) of an RNP scaffold that directly impacts chromatin structure

Many years ago, after publication of Fackelmayer’s initial work linking SAF-A to XIST RNA (Helbig and Fackelmayer 2003), we considered it highly likely that SAF-A was the tether that bound XIST RNA to chromosomal DNA. As we began to study SAF-A in human cells, the paper by Hasegawa et al. (2010) showed more direct evidence of an interaction between SAF-A and XIST RNA, and in the mouse cells studied, appeared to prove the straightforward model of SAF-A as a single-molecule bridge required to tether XIST RNA to chromosomal DNA (see Models, Fig. 6A). While we also found SAF-A depletion fully released XIST RNA in this particular mouse tumor cell line, our prior study focused on XIST RNA (Kolpa et al. 2016) showed different results and greater biological complexity in other cell lines, including normal primary somatic cells. Other evidence indicates various sources of tumor cells often have mis-localized XIST RNA (Pageau et al. 2007), indicating they may have a compromised “scaffold”. XIST RNA has been heavily studied for its role in recruiting repressive histone modifications to chromatin in cis, and thus the simpler model (Fig. 6A) of XIST RNA being tethered to chromatin in order to recruit histone modifiers (which in turn silence genes) was reasonable. Suffice it to say, our perspective of how this pre-eminent lncRNA, XIST, acts to modify a euchromatic chromosome into a heterochromatic Barr Body has evolved substantially.

XIST RNA certainly functions by triggering histone modifications as many labs have studied, but it may also change chromosome architecture at a higher level. We have hypothesized that XIST RNA may counter physical effects of euchromatin-associated “C_0_T-1 RNAs” that maintain decondensed open chromatin (Hall et al. 2014). This more speculative suggestion is now bolstered by a series of experiments demonstrating that this subset (~ 15%) of nuclear RNA (C_0_T-1 RNA) is with an RNP “scaffold” that retains structural integrity independent of chromatin (Creamer et al. 2021). While RNase treatment has long been known to disrupt nuclear and chromosome structure (Caudron-Herger et al. 2011; Hall et al. 2014; Nickerson et al. 1989) whether this is an indirect, non-specific effect remained an important question. Recent findings now support that these effects of RNase reflect a direct contribution of long nascent RNAs to physical architecture of chromosome territories, as schematically modeled in Fig. 6B. These collective findings suggest the important implication and new concept that the unexplained mass of largely non-coding and repeat-rich RNA the genome produces (including pre-mRNA introns and diverse lncRNAs) may serve essentially as a physical component of the chromosome territory (Creamer et al. 2021). Each long nascent transcript, even as it emanates from chromatin, has the capacity to bind many proteins, and thus could platform complex RNP structures. On a larger scale, this has been shown to be the case for the Neat1 lncRNA, which binds numerous proteins as its transcribed, that nucleate to form paraspeckle bodies (Smith et al. 2020). SAF-A is one important and interesting protein that broadly binds RNAs on chromatin, but it is likely one of a number of such factors that bind long chromatin-associated RNAs and contribute to complex networks (Fig. 6B).

This broader concept informs how we interpret results here to now “make sense” of what were initially perplexing and seemingly contradictory results; our endeavor to understand this began with our earlier results that confounded expectations that human SAF-A was required to tether XIST RNA. Kolpa et al. (2016) earlier demonstrated one source of complication to this biology: dependence on SAF-A for XIST RNA localization was clearly evidenced in a particular mouse tumor line but had no discernible effect in normal human cell types examined. However, in normal cells deletion or mutation of SAF-A’s DNA binding domain fully delocalized XIST RNA, consistent with a potential dominant negative effect. As described above, by examining more broadly effects of SAF-A mutants on C_0_T-1 RNA, and by examining C_0_T-1 RNA distribution relative to SAF-A mutants or endogenous protein, results strongly support that SAF-A mutants have dominant negative effects on RNA-chromatin association. Importantly, results show this disruption of RNA by SAF-A mutants is concomitant with grossly abnormal chromatin condensation. As seen with XIST RNA, SAF-A depletion had no discernible effect on C_0_T-1 RNA localization to euchromatin. In contrast, for all three SAF-A mutants, C_0_T-1 RNA becomes delocalized into DAPI “holes” (regions of low DNA density), and the RNA is apparently displaced along with mutant SAF-A (lacking DNA binding) or endogenous SAF-A displaced by the mutant SAF-A. These findings strengthen the evidence that RNA itself is a key determinant of physical chromosome structure, and also highlight the importance of considering a mutant protein’s effects on RNA in interpreting its role in chromatin.

The precise mechanism whereby SAF-A mutants disrupt RNA distribution and impact chromosome condensation remains to be determined, although we provide initial evidence that multiple chromatin-associated factors will be impacted. While not examined in depth, we demonstrate that a SAF-A mutant disrupts the distribution of certain other RNP proteins, such as FUS, further supporting a dominant negative effect on other potential scaffold factors. While we do not rule out that depletion of SAF-A alone could have some compromising effect, disruption of the more complex RNP scaffold associated with chromatin likely explains the pronounced effects on SAF-A mutants. Given that a large component of C_0_T-1 RNA is pre-mRNA undergoing synthesis, it will be of interest to determine whether SAF-A disrupts the RNA polymerase II (Pol II) transcriptional machinery. SAF-A has been reported to interact with RNA Pol II although some evidence indicates it is not required to maintain ongoing transcription (Kim and Nikodem 1999; Kukalev et al. 2005; Vizlin-Hodzic et al. 2011). SAF-A also has a low-complexity domain that potentially forms phase separated structures. Thus, it is possible that SAF-A mutants somehow interfere with RNA Pol II machinery that is also thought to function in thousands of small condensates (Guo et al. 2019).

In sum, chromosome-bound RNAs are generally not merely tethered by a bridging protein but are intimately intertwined in a complex RNA–protein structure that regulates nuclear architecture at a macro-level (Fig. 6).

## Materials and methods

### Cloning

SAF-A cDNA was cloned into pEGFP-N1 vector (Clontech) and the ΔRGG and G29A mutants made by PCR-based mutagenesis. Dominant negative C280-GFP was cloned into pEGFP-NLS (Clontech) after PCR amplification. Flag tags were added by restriction digest to cut GFP from pEGFP-N1and ligating with annealed oligo adaptor. For stable lines, Flag-tagged constructs were cloned into pcDNA/FRT/TO (Invitrogen). Mutagenesis to SAF-A siRNA target sites was done by Genscript: GGC → GGA (Glycine), TCG → TCA (Serine), GTT → GTA (Valine), and GTT → GTA (Valine). See Supplemental Experimental Procedures for primer sequences.

### Primers

ΔRGG: Fwd: 5′-TCCGTTAAACTGGTTCTTGCCACTCTTA-3′.

Rev: 5′-TCCGGCGGTGGAGGAAGTGGTGGAATC-3′.

G29A: Fwd: 5′-ACGCCTTTCTGACAAGGCCCTCAAGGCCGAGCTC-3′.

Rev: 5′-GAGCTCGGCCTTGAGGGCCTTGTCAGAAAGGCGT-3′.

C280: Fwd: 5′-AGATCGA ATTCTGTTTAAGAAGCAAATG GCAGAT-3′.

Rev: 5′-AGATCGGATCCGAATAATATCCTTGGTGATA ATGC-3′.

Flag oligo adaptors:

sense: 5′-AGCTTGATTACAAGGATGACGACGATAAGATCTGAGCGGCCGCGGTAC-3′.

antisense: 5′-CGCGGCCGCTCAGATCTTATCGTCGTCATCCTTGTAATCA-3′.

### Cell culture and fixation

We previously demonstrated clear phenotypic differences, with respect to the localization of XIST RNA to chromatin and the reliance on SAF-A between different cell types (Kolpa et al. 2016); transformed cells exhibit heterogeneity not only between tissue types, but also cell types, cell lines, and individual cells. Thus, we used normal human fibroblasts whenever possible. Tig-1 (Coriell) normal female fibroblasts and GM11687 mouse–human hybrid cells were maintained according to supplier’s instructions. Cell synchronization: cells were arrested in Nocodazole (100 ng/mL) for 5 h and collected by mitotic shakeoff prior to transfection. For analysis of mitotics, cells were trypsinized and diluted in cold 1 × PBS then cytospun at 5000 RPM onto coverslips as described (Johnson et al., 1991). RNase: we permeabilized unfixed cells on coverslips with 0.1% Triton-X in CSK buffer (4 °C for 3 min) then treated with 5 µL/mL DNase-free RNase (Roche #11119915001) or RNasin Plus RNase inhibitor (Promega), in CSK (10–30 min, 37 °C). Our standard cell fixation protocols have been previously described (Byron et al., 2013). Briefly, cells were grown on glass coverslips and extracted on ice in cytoskeletal buffer, 0.5% Triton X-100, and vanadyl ribonucleoside complex for 3–5 min. Cells were fixed at room temperature for 10 min in 4% paraformaldehyde and stored at 4 °C in 70% EtOH.

### siRNA and plasmid transfections

For RNAi, cells (70–80% confluent) were transfected with SMARTpool siRNAs and DharmaFECT 1 transfection reagent (GE Dharmacon) according to manufacturer’s instructions and fixed after 72 h. siGLO siRNA (GE Dharmacon) was used as a control which had no impact on RNA localization. For plasmid transfections, 2–4 μg of DNA was mixed with Lipofectamine 2000 (Invitrogen) according to manufacturer’s instructions and added to 80–90% confluent cells in a 6-well plate. Cells were fixed and assayed 24, 48, or 72 h after transfection.

### Immunofluorescence

As previously described (Byron et al. 2013), coverslips were incubated in primary antibody diluted in 1% BSA, 1 × PBS for 1 h at 37 °C. They were then washed and immunodetected with a 1:500 dilution of fluorescent conjugated anti-mouse or -rabbit antibody in 1 × PBS with 1% BSA. Prior to some SAF-A antibody stains, we treated cells for antigen retrieval using established procedures by incubating coverslips in 10 mM citric acid and 0.05% tween at 100 °C for 20–40 min. This method is commonly used to expose epitopes in clinical tissue samples, and allowed us to visualize the enriched layer of endogenous SAF-A on the Barr body, previously seen only after a matrix prep (Helbig and Fackelmayer 2003). For protein/RNA detection, antibody stains were done with added 1 U/µl of RNasin Plus RNase inhibitor (Promega) and signals fixed for 10 min in 4% paraformaldehyde prior to hybridization. Coverslips were counterstained with DAPI and mounted with Vectashield (Vector Laboratories) for imaging. Antibodies used were anti-SAF-A (Abcam ab20666 and ab10297), anti-FUS (Bethyl Labs), anti-hnRNP C (Abcam ab97541), anti-SafB1 (Abcam ab8060), anti-NuMA (Abcam ab36999), anti-Lamin B1 (Abcam ab16048 and Santa Cruz sc-6217).

### Fluorescence in situ hybridizations

RNA hybridization was performed under non-denaturing conditions as previously described (Byron et al. 2013). C_0_T-1 DNA (Roche) was nick translated with either biotin-11-dUTP or digoxigenin-16-dUTP (Roche). Hybridizations were done overnight at 37 °C in 2 × SSC, 1 U/μl of RNasin Plus RNase inhibitor (Promega), and 50% formamide with 2.5 µg/ml DNA probe. Cells were washed with 50% formamide/2 × SSC at 37 °C for 20 min, 2 × SSC at 37 °C for 20 min, 1 × SSC at RT for 20 min, and 4 × SSC at RT for 1 min. Detection was with antidigoxigenin or fluorescein-conjugated avidin in 1% BSA/4 × SSC for 1 h at 37 °C. Three 10 min washes were done in 4 × SSC, 4 × SSC with 0.1% Triton, and 4 × SSC at RT in the dark. Coverslips were counterstained with DAPI and mounted with Vectashield (Vector Laboratories).

### Microscopy and image analysis

Experiments were performed a minimum of three times (all independent transient transfection experiments), and a minimum of 50 and typically 150 cells were scored in each experiment. To account for potential effects of protein over-expression, transiently transfected cells were scored according to whether they expressed dim, moderate, bright, and very bright SAF-A-GFP levels, and the grossly overexpressing cells were eliminated. Digital imaging and analysis was performed using an Axiovert 200 microscope (Carl Zeiss, Inc.) with a 100 × NA 1.4 Plan-Apochromat objective and multi-bandpass dichroic and emission filter sets (model 83,000; Chroma Technology Corp) set up in a wheel to prevent optical shift. We used AxioVision software (Carl Zeiss, Inc.) and an Orca-ER camera (Hamamatsu Photonics). A narrow bandpass fluorescein filter was inserted to eliminate cross talk between channels. Super-resolution 3D-SIM images were acquired on a DeltaVision OMX V4 (GE Healthcare) equipped with a 60x/1.42 NA PlanApo oil immersion lens (Olympus), 405, 488, 568, and 642 nm solid state lasers and sCMOS cameras (pco.edge). Image stacks of 7–9 µm with 0.125 µm thick *z*-sections and 15 images per optical slice (3 angles and 5 phases) were acquired using immersion oil with a refractive index of 1.518. Images were reconstructed using Wiener filter settings of 0.001 and optical transfer functions (OTFs) measured specifically for each channel with SoftWoRx 6.1.3 (GE Healthcare) to obtain super-resolution images with a two-fold increase in resolution both axially and laterally. Images from different color channels were registered using parameters generated from a gold grid registration slide (GE Healthcare) and SoftWoRx 6.5.2 (GE Healthcare). Huygens was used to measure Chr 4 territory voxel volumes, Fiji to quantify SAF-A signal intensity (before and after SAF-A RNAi) in individual nuclei, and when necessary, Photoshop (Adobe) or Fiji to enhance images for brightness and contrast.

### GFP and flag tags

We have noted that SAF-A binding to chromatin appears to be affected by the type of tag fused to the protein. Most of this was discovered during work done for XIST RNA localization studies (Kolpa et al. 2016). SAF-A mutants with a C-terminal GFP-tag caused a dominant mis-localization of XIST RNA in normal cells, whereas the smaller C-terminal Flag-tag did not. It also affected a higher percentage of cells in transformed cultures. We also discovered that wild-type SAF-A with a C-terminal GFP also remained bound to chromosomes in mitosis whereas endogenous SAF-A and Flag-tagged SAF-A did not. When GFP is fused to the N-terminus of SAF-A, near the SAP (DNA binding) domain, the mutants were more disruptive to XIST RNA localization than when GFP was used to the C-terminus, near the RGG (RNA binding) domain. All of this suggests the large GFP-tag was impacting specific SAF-A domains.

## Supplementary Information

Below is the link to the electronic supplementary material.

Supplementary file1 Supplemental figure 1: Endogenous SAF-A is enriched on the Xi, released during mitosis, binds RNAs, but is not necessary for RNA localization. For all images: color channels are separated in black and white. Scale bars 5 μm. Cell types: Normal human fibroblasts (Tig-1). **﻿A** SAF-A begins releasing from chromatin in an early prophase cell (close up of region in the outline) but the residual SAF-A is clearly associated with chromatin before release (SIM image). **B** Antibody labeling for endogenous SAF-A sometimes appears diminished over heterochromatin, including the Barr body (arrow). **C** With antigen retrieval methods to expose embedded epitopes, SAF-A labeling increases over heterochromatin, including enrichment over the Barr body (defined by XIST RNA localization). **D** Both C_0_T-1 RNA and SAF-A are released from chromatin at mitosis (metaphase cell). **E**–**F** C_0_T-1 RNA remains bound to chromatin before (**E**) and after (**F**) SAF-A RNAi, and DAPI DNA morphology remains unaffected (TIF 24155 kb)

Supplementary file2 Supplemental figure 2: C280 SAF-A displaces specific nuclear proteins and hnRNAs to disrupt DNA structure. For all images: color channels are separated in black and white. Scale bars 5 μm. Cell types: Normal human fibroblasts (Tig-1). **A**  Additional examples of C280-GFP effects on NuMA. Neighboring cells lacking C280-GFP are normal controls. **B** Additional examples of the lack of effect of C280-GFP on LaminB1 nuclear distribution. Neighboring cells lacking C280-GFP are normal controls. **C** More examples of C280-GFP effects on FUS. **D** Additional example of the pattern seen for how C280-GFP affects hnRNP C. **E** Additional examples of C280-GFP effects on SAFB1 (TIF 30314 kb)

Supplementary file3 Supplemental figure 3: SAF-A mutants displace C_0_T-1 RNA and alter DNA morphology For all images: color channels are separated in black and white. Scale bars 5 μm. Cell types: Normal human fibroblasts (Tig-1) & mouse/human hybrid cells with human chromosome 4 (Hybrid). **A** Two neighboring fibroblast nuclei, with (top) and without (bottom) expression of the G29A SAF-A mutant. The top cell shows changes to mutant protein distribution, C_0_T-1 RNA distribution and DNA morphology. **B** A linescan histogram of all color channels in the path indicated in image (A). **C** More examples of G29A-GFP effects on C_0_T-1 RNA. Neighboring cells lacking G29A-GFP are normal controls. **D** More examples of DRGG-GFP effects on C_0_T-1 RNA in hybrid cells. Neighboring cell lacking DRGG-GFP is a normal control. **E** More examples of C280-GFP effects on C_0_T-1 RNA (TIF 29306 kb)

## Data Availability

No novel datasets were generated as part of this study.

## Code availability

No novel code was used as part of this study.

## References

[CR1] Brockdorff N, Bowness JS, Wei G (2020). Progress toward understanding chromosome silencing by Xist RNA. Genes Dev.

[CR2] Brown CJ, Hendrich BD, Rupert JL, Lafreniere RG, Xing Y, Lawrence J, Willard HF (1992). The human XIST gene: analysis of a 17 kb inactive X-specific RNA that contains conserved repeats and is highly localized within the nucleus. Cell.

[CR3] Byron M, Hall LL, Lawrence JB (2013). A multifaceted FISH approach to study endogenous RNAs and DNAs in native nuclear and cell structures. Curr Protoc Hum Genet.

[CR4] Carter KC, Taneja KL, Lawrence JB (1991). Discrete nuclear domains of poly(A) RNA and their relationship to the functional organization of the nucleus. J Cell Biol.

[CR5] Caudron-Herger M, Muller-Ott K, Mallm JP, Marth C, Schmidt U, Fejes-Toth K, Rippe K (2011). Coding RNAs with a non-coding function: maintenance of open chromatin structure. Nucleus.

[CR6] Clemson CM, McNeil JA, Willard HF, Lawrence JB (1996). XIST RNA paints the inactive X chromosome at interphase: evidence for a novel RNA involved in nuclear/chromosome structure. J Cell Biol.

[CR7] Creamer KM, Lawrence JB (2017). XIST RNA: a window into the broader role of RNA in nuclear chromosome architecture. Philos Trans R Soc Lond B Biol Sci.

[CR8] Creamer KM, Kolpa HJ, Lawrence JB (2021). Nascent RNA scaffolds contribute to chromosome territory architecture and counter chromatin compaction. Mol Cell.

[CR9] Durkin A, Albaba S, Fry AE, Morton JE, Douglas A, Beleza A, Williams D, Volker-Touw CML, Lynch SA, Canham N (2020). Clinical findings of 21 previously unreported probands with HNRNPU-related syndrome and comprehensive literature review. Am J Med Genet Part A.

[CR10] Fackelmayer FO, Dahm K, Renz A, Ramsperger U, Richter A (1994). Nucleic-acid-binding properties of hnRNP-U/SAF-A, a nuclear-matrix protein which binds DNA and RNA in vivo and in vitro. Eur J Biochem.

[CR11] Fan H, Lv P, Huo X, Wu J, Wang Q, Cheng L, Liu Y, Tang QQ, Zhang L, Zhang F (2018). The nuclear matrix protein HNRNPU maintains 3D genome architecture globally in mouse hepatocytes. Genome Res.

[CR12] Gendrel AV, Heard E (2014). Noncoding RNAs and epigenetic mechanisms during X-chromosome inactivation. Annu Rev Cell Dev Biol.

[CR13] Gohring F, Fackelmayer FO (1997). The scaffold/matrix attachment region binding protein hnRNP-U (SAF-A) is directly bound to chromosomal DNA in vivo: a chemical cross-linking study. Biochemistry.

[CR14] Guo YE, Manteiga JC, Henninger JE, Sabari BR, Dall'Agnese A, Hannett NM, Spille JH, Afeyan LK, Zamudio AV, Shrinivas K (2019). Pol II phosphorylation regulates a switch between transcriptional and splicing condensates. Nature.

[CR15] Hacisuleyman E, Goff LA, Trapnell C, Williams A, Henao-Mejia J, Sun L, McClanahan P, Hendrickson DG, Sauvageau M, Kelley DR (2014). Topological organization of multichromosomal regions by the long intergenic noncoding RNA Firre. Nat Struct Mol Biol.

[CR16] Hall LL, Smith KP, Byron M, Lawrence JB (2006). Molecular anatomy of a speckle. Anat Rec A Discov Mol Cell Evol Biol.

[CR17] Hall LL, Carone DM, Gomez AV, Kolpa HJ, Byron M, Mehta N, Fackelmayer FO, Lawrence JB (2014). Stable C0T–1 repeat RNA is abundant and is associated with euchromatic interphase chromosomes. Cell.

[CR18] Hasegawa Y, Brockdorff N, Kawano S, Tsutui K, Tsutui K, Nakagawa S (2010). The matrix protein hnRNP U is required for chromosomal localization of Xist RNA. Dev Cell.

[CR19] Helbig R, Fackelmayer FO (2003). Scaffold attachment factor A (SAF-A) is concentrated in inactive X chromosome territories through its RGG domain. Chromosoma.

[CR20] Johnson CV, Singer RH, Lawrence JB (1991). Fluorescent detection of nuclear RNA and DNA: implications for genome organization. Methods Cell Biol.

[CR21] Jurica MS, Licklider LJ, Gygi SR, Grigorieff N, Moore MJ (2002). Purification and characterization of native spliceosomes suitable for three-dimensional structural analysis. RNA.

[CR22] Kiledjian M, Dreyfuss G (1992). Primary structure and binding activity of the hnRNP U protein: binding RNA through RGG box. EMBO J.

[CR23] Kim MK, Nikodem VM (1999). hnRNP U inhibits carboxy-terminal domain phosphorylation by TFIIH and represses RNA polymerase II elongation. Mol Cell Biol.

[CR24] Kolpa HJ, Fackelmayer FO, Lawrence JB (2016). SAF-A requirement in anchoring XIST RNA to chromatin varies in transformed and primary cells. Dev Cell.

[CR25] Kukalev A, Nord Y, Palmberg C, Bergman T, Percipalle P (2005). Actin and hnRNP U cooperate for productive transcription by RNA polymerase II. Nat Struct Mol Biol.

[CR26] Loda A, Heard E (2019). Xist RNA in action: past, present, and future. PLoS Genet.

[CR27] Nakagawa S, Prasanth KV (2011). eXIST with matrix-associated proteins. Trends Cell Biol.

[CR28] Nickerson J (2001). Experimental observations of a nuclear matrix. J Cell Sci.

[CR29] Nickerson JA, Krochmalnic G, Wan KM, Penman S (1989). Chromatin architecture and nuclear RNA. Proc Natl Acad Sci USA.

[CR30] Nozawa RS, Boteva L, Soares DC, Naughton C, Dun AR, Buckle A, Ramsahoye B, Bruton PC, Saleeb RS, Arnedo M (2017). SAF-A regulates interphase chromosome structure through oligomerization with chromatin-associated RNAs. Cell.

[CR31] Pageau GJ, Hall LL, Lawrence JB (2007). BRCA1 does not paint the inactive X to localize XIST RNA but may contribute to broad changes in cancer that impact XIST and Xi heterochromatin. J Cell Biochem.

[CR32] Puvvula PK, Desetty RD, Pineau P, Marchio A, Moon A, Dejean A, Bischof O (2014). Long noncoding RNA PANDA and scaffold-attachment-factor SAFA control senescence entry and exit. Nat Commun.

[CR33] Saitoh N, Spahr CS, Patterson SD, Bubulya P, Neuwald AF, Spector DL (2004). Proteomic analysis of interchromatin granule clusters. Mol Biol Cell.

[CR34] Sharp JA, Perea-Resa C, Wang W, Blower MD (2020). Cell division requires RNA eviction from condensing chromosomes. J Cell Biol.

[CR35] Smith KP, Hall LL, Lawrence JB (2020). Nuclear hubs built on RNAs and clustered organization of the genome. Curr Opin Cell Biol.

[CR36] Spector DL, Lamond AI (2011). Nuclear speckles. Cold Spring Harbor Perspect Biol.

[CR37] Sunwoo H, Colognori D, Froberg JE, Jeon Y, Lee JT (2017). Repeat E anchors Xist RNA to the inactive X chromosomal compartment through CDKN1A-interacting protein (CIZ1). Proc Natl Acad Sci USA.

[CR38] Thandapani P, O’Connor TR, Bailey TL, Richard S (2013). Defining the RGG/RG motif. Mol Cell.

[CR39] Vizlin-Hodzic D, Runnberg R, Ryme J, Simonsson S, Simonsson T (2011). SAF-A forms a complex with BRG1 and both components are required for RNA polymerase II mediated transcription. PLoS One.

[CR40] Xiao R, Tang P, Yang B, Huang J, Zhou Y, Shao C, Li H, Sun H, Zhang Y, Fu XD (2012). Nuclear matrix factor hnRNP U/SAF-A exerts a global control of alternative splicing by regulating U2 snRNP maturation. Mol Cell.

[CR41] Ye J, Beetz N, O’Keeffe SO, Tapia JC, Macpherson L, Chen WV, Bassel-Duby R, Olson EN, Maniatis T (2015). hnRNP U protein is required for normal pre-mRNA splicing and postnatal heart development and function. Proc Natl Acad Sci USA.

[CR42] Zhou Z, Licklider LJ, Gygi SP, Reed R (2002). Comprehensive proteomic analysis of the human spliceosome. Nature.

