# Supplementary figures and images for: SAF-A mutants disrupt chromatin structure through dominant negative effects on RNAs associated with chromatin
Source: Mamm Genome. 2021 Dec 2;33(2):366–81. doi: 10.1007/s00335-021-09935-8 (PMC9114059; doi:10.1007/s00335-021-09935-8)

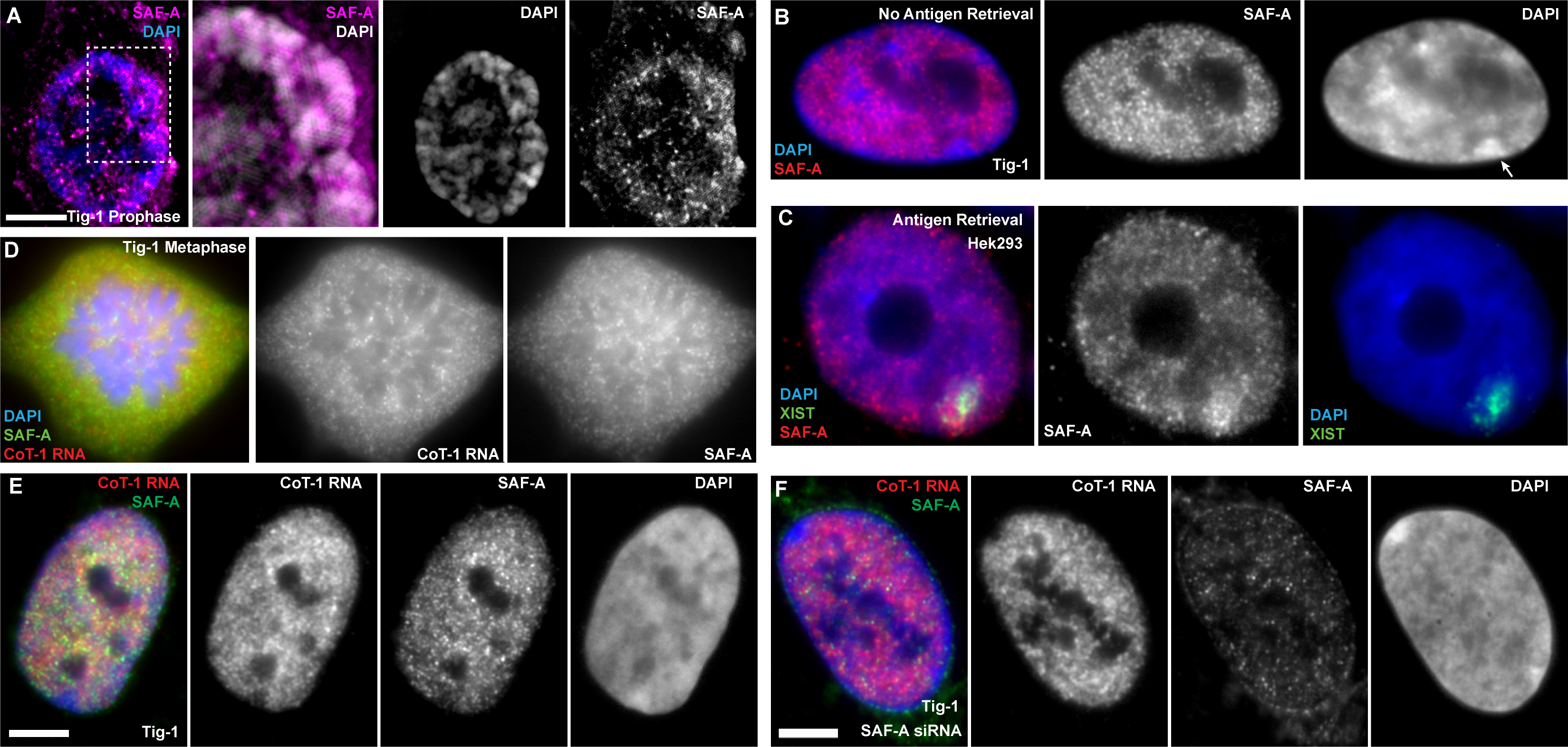

Supplement: Supplementary file 1 — Supplementary file1 Supplemental figure 1: Endogenous SAF-A is enriched on the Xi, released during mitosis, binds RNAs, but is not necessary for RNA localization. For all images: color channels are separated in black and white. Scale bars 5 μm. Cell types: Normal human fibroblasts (Tig-1). ﻿A SAF-A begins releasing from chromatin in an early prophase cell (close up of region in the outline) but the residual SAF-A is clearly associated with chromatin before release (SIM image). B Antibody labeling for endogenous SAF-A sometimes appears diminished over heterochromatin, including the Barr body (arrow). C With antigen retrieval methods to expose embedded epitopes, SAF-A labeling increases over heterochromatin, including enrichment over the Barr body (defined by XIST RNA localization). D Both C0T-1 RNA and SAF-A are released from chromatin at mitosis (metaphase cell). E–F C0T-1 RNA remains bound to chromatin before (E) and after (F) SAF-A RNAi, and DAPI DNA morphology remains unaffected (TIF 24155 kb) [file 335_2021_9935_MOESM1_ESM.tif]

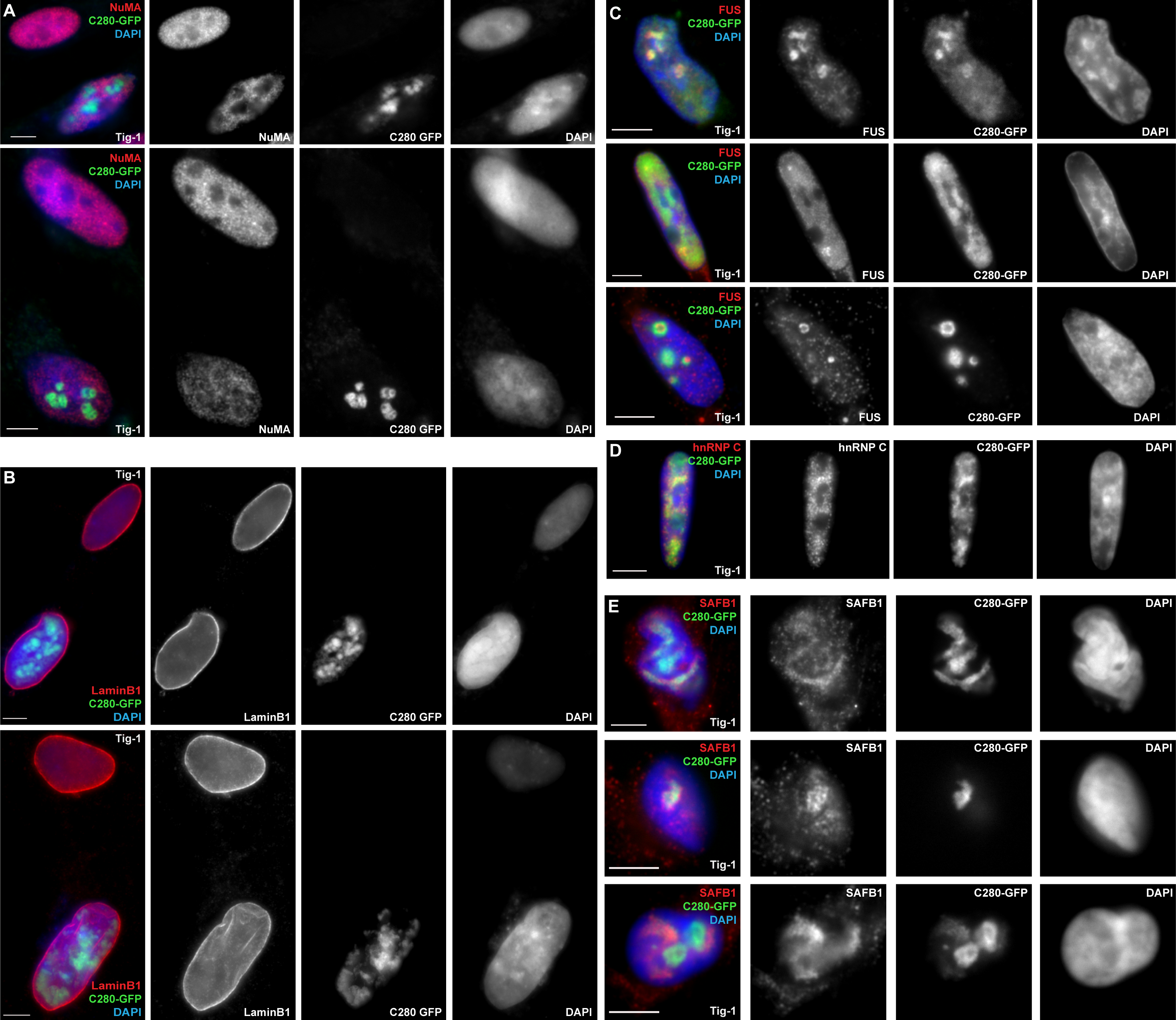

Supplement: Supplementary file 2 — Supplementary file2 Supplemental figure 2: C280 SAF-A displaces specific nuclear proteins and hnRNAs to disrupt DNA structure. For all images: color channels are separated in black and white. Scale bars 5 μm. Cell types: Normal human fibroblasts (Tig-1). A Additional examples of C280-GFP effects on NuMA. Neighboring cells lacking C280-GFP are normal controls. B Additional examples of the lack of effect of C280-GFP on LaminB1 nuclear distribution. Neighboring cells lacking C280-GFP are normal controls. C More examples of C280-GFP effects on FUS. D Additional example of the pattern seen for how C280-GFP affects hnRNP C. E Additional examples of C280-GFP effects on SAFB1 (TIF 30314 kb) [file 335_2021_9935_MOESM2_ESM.tif]

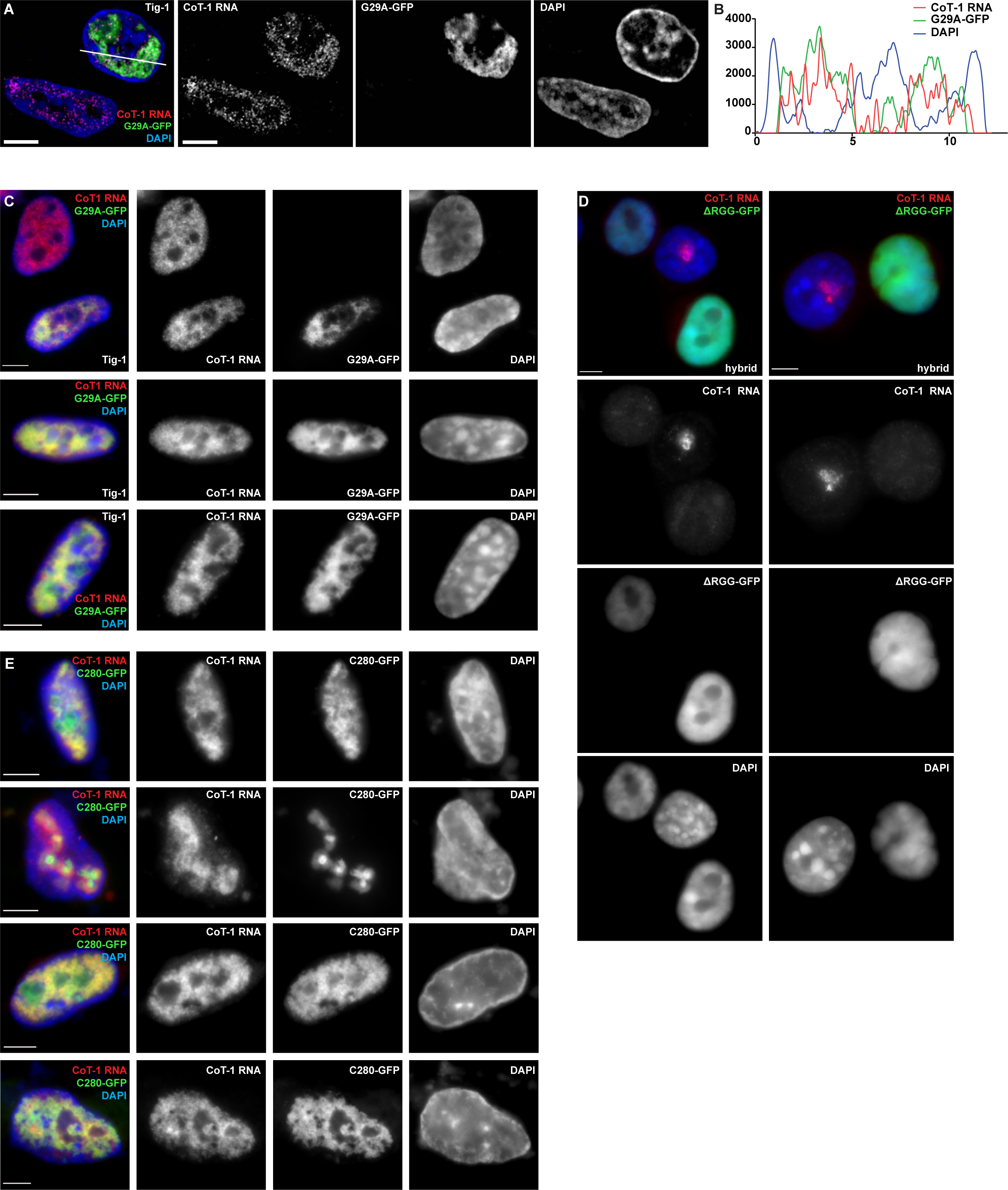

Supplement: Supplementary file 3 — Supplementary file3 Supplemental figure 3: SAF-A mutants displace C0T-1 RNA and alter DNA morphology For all images: color channels are separated in black and white. Scale bars 5 μm. Cell types: Normal human fibroblasts (Tig-1) & mouse/human hybrid cells with human chromosome 4 (Hybrid). A Two neighboring fibroblast nuclei, with (top) and without (bottom) expression of the G29A SAF-A mutant. The top cell shows changes to mutant protein distribution, C0T-1 RNA distribution and DNA morphology. B A linescan histogram of all color channels in the path indicated in image (A). C More examples of G29A-GFP effects on C0T-1 RNA. Neighboring cells lacking G29A-GFP are normal controls. D More examples of DRGG-GFP effects on C0T-1 RNA in hybrid cells. Neighboring cell lacking DRGG-GFP is a normal control. E More examples of C280-GFP effects on C0T-1 RNA (TIF 29306 kb) [file 335_2021_9935_MOESM3_ESM.tif]
